# Dielectric Relaxation Characteristics of Epoxy Resin Modified with Hydroxyl-Terminated Nitrile Rubber

**DOI:** 10.3390/molecules25184128

**Published:** 2020-09-10

**Authors:** Chi Chen, Qing Sun, Chuang Wang, Yue Bu, Jiawei Zhang, Zongren Peng

**Affiliations:** 1 School of Electrical Engineering, Xi’an University of Technology, Xi’an 710048, China; chenchi0129@stu.xjtu.edu.cn (C.C.); 2180421168@stu.xaut.edu.cn (Q.S.); buyue@xaut.edu.cn (Y.B.); zhangjiawei@xaut.edu.cn (J.Z.); 2 State Key Laboratory of Electrical Insulation and Power Equipment, Xi’an Jiaotong University, Xi’an 710049, China; zrpeng@xjtu.edu.cn

**Keywords:** HTBN, epoxy resin, relaxation characteristics, interfacial polarization

## Abstract

Utilizing liquid rubber to toughen epoxy resin is one of the most mature and promising methods. However, the dielectric relaxation characteristics of the epoxy/liquid rubber composites have not been studied systematically, while the relaxation behaviours are a critical factor for both micro and macro properties. In this paper, hydroxyl-terminated liquid nitrile rubber (HTBN) is employed to reinforce a kind of room-temperature-cured epoxy resin. The dielectric spectrum is measured and analysed. Results show that two relaxation processes are introduced in the binary composites. The α relaxation of HTBN shows a similar temperature dependence with the β relaxation of epoxy resin. The interfacial polarization leads to an increase of complex permittivity, which reaches its maximum at 70 °C. In addition, affected by interfacial polarization, the thermionic polarization is inhibited, and the samples with filler ratios of 15% and 25% show lower DC-conductivity below 150 °C. In addition, the α relaxation and thermionic polarization of epoxy resin obey the Vogel‒Fulcher‒Tammann law, while the interfacial polarization and DC-conductivity satisfy with the Arrhenius law. Furthermore, the fitting results of the Vogel temperature of α relaxation, glass transition temperature, apparent activation energy of interfacial polarization and DC-conductivity all decline with HTBN content. These results can provide a reference and theoretical guidance for the assessment of dielectric properties and the improvement of the formulation of liquid-rubber-toughened epoxy resin.

## 1. Introduction

Liquid rubber, such as hydroxyl-terminated liquid nitrile rubber (HTBN), is one kind of efficient toughener for epoxy resin owing to the simple operation process [1,2]. Epoxy resin/liquid rubber composites and their nano modified composites have been applied to electrical packaging and power equipment, so the dielectric properties must be of comparable concern [3,4]. Liquid rubber uniformly disperses in the matrix in the form of discrete phases, and the dielectric properties are changed, which is attributed to the variation of relaxation behaviours [5,6]. So far, various kinds of liquid rubber have been adopted to toughen epoxy resin. Previous studies mainly focus on the toughening mechanism from the perspectives of kinetics and morphology [7,8,9,10] or evaluate the dielectric properties according to parameters such as permittivity and electrical breakdown strength [11], but the relaxation characteristics have not been systematically discussed and the mechanism of influence on dielectric properties is obscure.

Zhou and his co-workers modified epoxy resin with hydroxyl-terminated polybutadiene (HTPB) liquid rubber [11], and the interfacial polarization is mentioned, but its relaxation characteristics are excluded. The influence of carboxyl-terminated polybutadiene liquid rubber (CTPB) has also been studied [4]. The ternary h-BN/CTPB/epoxy shows better dielectric and thermal properties, but only the dielectric properties at room temperature are analysed. Two new relaxation processes have been found in epoxy composites modified using HTBN [5]. However, the exploration of interfacial polarization and ionic conduction is unspecific. In recent research [6], by comparison of the relaxation properties of the epoxy composites toughened using different liquid rubber, we found that a lower relative permittivity and dielectric loss of the epoxy/liquid rubber composites could be achieved by reducing the polarity of the liquid rubber filler. The dielectric behaviours at different frequencies and temperatures can characterise the orientation polarization of dipoles, the motion of groups or segments, the transport characteristics of ions and some other parameters, which can establish the relationship between the microstructure and macroscopic electrical properties [12]. Therefore, it is necessary to carry out further studies to analyse the relaxation characteristics.

In this research, based on the application of insulation packaging and interface buffering, a kind of room-temperature-cured epoxy resin is adopted, and HTBN is employed, owing to its better improvement of mechanical properties. The dielectric spectroscopy of the composites is measured, and the Havriliak‒Negami equation is adopted to investigate the relaxation peaks of various relaxation processes. Through analysis, the influences of liquid rubber on the relaxation behaviours and dielectric characteristics are discussed.

## 2. Results and Discussion

Dielectric spectroscopy indicates the motion of molecule segments and groups, and the process of various polarization behaviours are also revealed. In this section, firstly, the variation of complex permittivity is discussed through the temperature spectroscopy. Then, an analysis method of dielectric relaxation is introduced. Finally, we give individually and systematically discussion to the dielectric relaxation characteristics of epoxy resin modified with HTBN.

### 2.1. Complex Permittivity Variation

**Figure 1:**
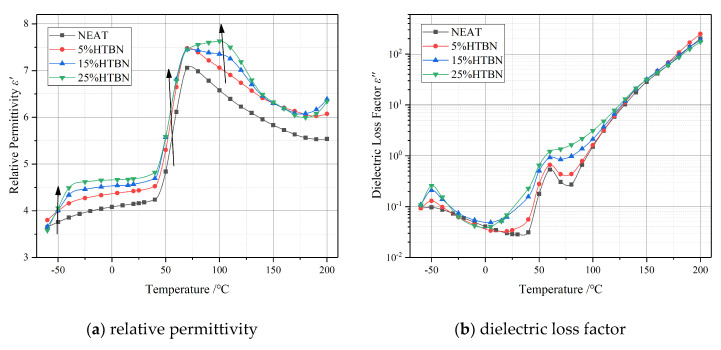
Temperature spectrum characteristics of epoxy resin with different hydroxyl-terminated liquid nitrile rubber (HTBN) contents at 50 Hz: (**a**) relative permittivity; (**b**) dielectric loss factor.

Temperature spectrum characteristics of complex permittivity with different liquid rubber contents at 50 Hz are shown in Figure 1, in which Figure 1a is the relative permittivity *ε*′, and Figure 1b is the dielectric loss factor *ε*″. The *ε*′ has a marked increase in composites toughened using liquid rubber. Furthermore, along the arrow in Figure 1a, the dielectric relaxations in the low- (from −60 °C to –10 °C), medium- (from 0 °C to 90 °C) and high-temperature region (from 90 °C to 200 °C) are also changed.

In the low-temperature region, *ε*″ increases firstly and then declines with the increase of temperature. A relaxation peak occurs at −50 °C which represents the secondary transition of the epoxy resin. The composites toughened using liquid rubber show a rather higher magnitude of relaxation peak compared to the pure epoxy resin. The positions of the relaxation peak with different filler contents are coincident, which should be a superposition of the orientation of a HTBN molecule (HTBN α relaxation) and the secondary transition in epoxy resin.

In the medium-temperature region, there is a rising on *ε*″ of the samples with HTBN, which is proportional to the filler contents. The relaxation peak appearing near 70 °C reflects the glass transition process formed by the movement of the segment of epoxy resin molecular chain, which is also called α relaxation. Moreover, the extension of the relaxation peak of the sample with liquid rubber is broadened, and the amplitude is elevated with the increase of filler contents. The non-uniformly distribution of conductivity and permittivity in polymers lead to space charge polarization (Maxwell‒Wagner polarization), also named interfacial polarization. The establishment time of interfacial polarization is equivalent to the dipole polarization. Therefore, the variation of *ε*″ at medium temperature region may be attributed to both the α relaxation and interfacial polarization [5,13].

The *ε*″ in the high temperature region presents a linear growth due to the impact of conduction. Moreover, it is worth noting that an intersection of *ε*″ appears at 150 °C. Therefore, the addition of HTBN also affects the ionic conduction process in the high-temperature region.

**Figure 2:**
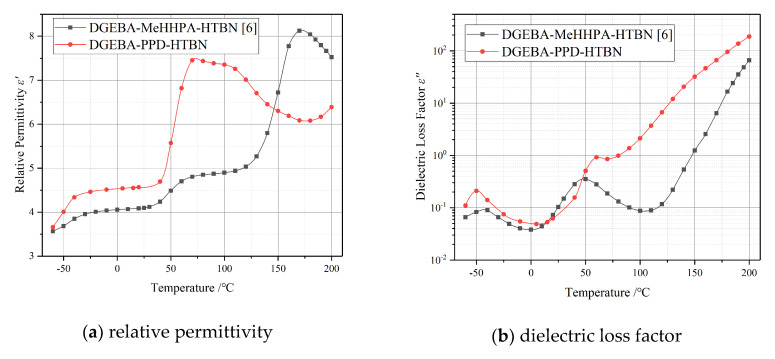
Temperature spectrum characteristics of epoxy resin/liquid rubber composites with different curing agents at 50 Hz: (**a**) relative permittivity; (**b**) dielectric loss factor. DGEBA-MeHHPA-HTBN: the epoxy resin is diglycidyl ether of bisphenol A (DGEBA), the curing agent is methylhexahydrophthalic anhydride(MeHHPA) and the toughening agent is HTBN. The glass transition temperature of DGEBA-MeHHPA-HTBN is 142 °C.

Two different curing agents, methylhexahydrophthalic anhydride (MeHHPA) and polyoxide propylene diamine (PPD), were used in the previous study [6] and this research respectively. Compared to the temperature spectrum characteristics in Figure 2, the polarization process of DGEBA-PPD-HTBN is stronger than that of DGEBA-MeHHPA-HTBN in the temperature range of 50 °C to 150 °C. This may be caused by the interfacial polarization, and the analysis of this part will be carried out in Section 2.4.

According to the variation of complex permittivity, the composites modified using HTBN indicate notable change, which is represented by the variation of relaxation polarization. Relaxation polarization has frequency-dependent and temperature-dependent properties. Multiple polarization processes may occur at a specific frequency or temperature, and the adjacent relaxation peaks will coincide. Therefore, the relaxation peaks should be decomposed when analysing the influence mechanism of HTBN on dielectric relaxation characteristics.

### 2.2. Dielectric Relaxation Characterization Method

Stem from the typical Debye function, a series of frequency-independent model functions of the complex permittivity, have been introduced [13]. As one of the most universal models, the Havriliak‒Negami equation (HN-equation) depicts a relaxation process with four parameters: relaxation time, relaxation strength, shape factor and symmetrical factor. The algebraic sum of the HN-equation represents the frequency characteristics of complex permittivity at a certain temperature. In addition, concerning the leading role of DC-conductivity at high temperature and low frequency, the model of complex permittivity is divided into two parts [14]. As shown in Equation (1), the first term in is the DC-conductivity loss, and the remaining two terms are the relaxation part.

(1) $$\varepsilon^{*}{}_{\mathrm{HN}}(\omega )=-i\left(\frac{\sigma_{\mathrm{dc}}}{\varepsilon_{0}\omega }\right)+\varepsilon_{\infty }+\sum_{k=1}^{n}\frac{\Delta \varepsilon_{k}}{{\left(1+{\left(i\omega \tau_{k}\right)}^{\beta_{k}}\right)}^{\gamma_{k}}},$$

where ε^*^_HN_(*ω*) is the complex permittivity, *ε*_0_ is the permittivity of the vacuum, *ω* is the angular frequency, *k* represents the number of relaxation behaviours, ∆*ε_k_* is the relaxation strength, *τ_k_* is the relaxation time, *β_k_* and *γ_k_* describe the symmetric and asymmetric broadening of the complex dielectric function, *σ*_dc_ is the DC-conductivity and *ε_∞_* is the permittivity when *f*→+∞.

Therefore, 4*n* + 2 parameters should be identified for a spectrum curve of complex permittivity. A kind of modified differential evolution algorithm is involved. In addition, the real part and image part of complex permittivity are both taken into consideration in Equation (2) to ensure accuracy. Finally, the parameter will be established by the least-square fitting.

(2) $$\sum_{i=1}^{N}\left[\varepsilon_{i}^{*}-\varepsilon_{i}^{*}{}_{\mathrm{HN}}(\omega_{i})\right]\to \min,$$

### 2.3. Analysis of Secondary Transition

According to the analysis of the temperature spectrum in Section 2.1, the distribution of relaxation processes is related to the applied temperature. Therefore, the relaxation characteristics of the sample modified using HTBN are discussed from the low-temperature region to the high-temperature region in the following section.

**Figure 3:**
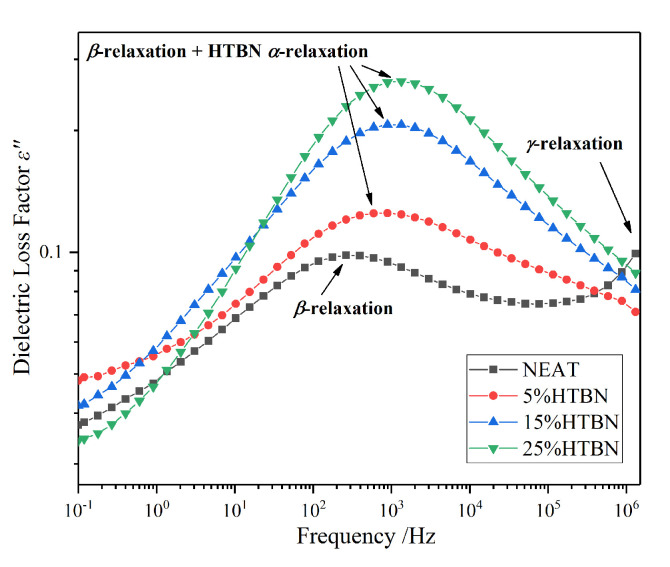
Frequency spectrum characteristics of dielectric loss factor with different HTBN contents at −40 °C.

The distribution of relaxation behaviours of four samples at −40 °C are demonstrated in Figure 3. The peaks of middle frequency reveal evident polarization processes, and the strength rises as the filler contents increase. The β relaxation caused by the movement of groups of epoxy resin appears near 200 Hz, while the distinct relaxation process in samples with HTBN is located near 10^3^ Hz.

The addition of HTBN will bring more hydroxyl groups into the epoxy matrix, which could enhance the β process. In addition, according to the temperature spectrum, the α relaxation of HTBN emerges at −50 °C. Therefore, the addition of HTBN not only intensifies the strength of β relaxation but also introduces a new relaxation polarization in the low-temperature region. The superposition of these two peaks contributes to the larger strength in samples with HTBN. Furthermore, with the increase of HTBN content, the number of HTBN molecular chains and hydroxyl groups per unit volume increases, leading to higher relaxation peaks.

In Figure 3, a high-frequency tail in pure epoxy resin represents the γ relaxation, and the relaxation peak of the pure epoxy resin may appear at a higher frequency above 10^6^ Hz. However, the dielectric loss factor declines at high frequency in all samples with HTBN. The γ relaxation is partly attributed to the local motions of dipoles associated with the unreacted epoxy groups and amine groups [15], and the flexible rubber molecule could increase the extent of reaction and reduce the unreached groups in the matrix [16]. Therefore, due to the introduction of the HTBN molecules, the γ relaxation process is restrained instead of moving to a higher frequency as in pure epoxy resin.

### 2.4. Analysis of Interfacial Polarization

**Figure 4:**
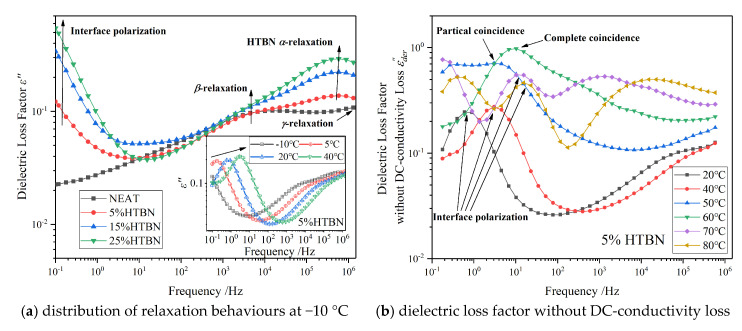
Frequency spectrum characteristics of dielectric loss factor: (**a**) distribution of relaxation behaviours at −10 °C; insert figure: temperature dependence of interfacial polarization in the sample with 5% HTBN content; (**b**) dielectric loss factor of the sample with 5% HTBN content without DC-conductivity loss in medium temperature region.

HTBN particles disperse in the epoxy matrix, and the interface forms between particles and matrix. Under the electric field, the charge will accumulate at the interface and cause the interfacial polarization. In low frequency from Figure 4a, the interfacial polarization comes to emerge at −10 °C and demonstrates the dependence of temperature along the arrow from the insert.

Due to the longer relaxation time, the relaxation peak will be covered by the DC-conductivity loss in the low-frequency region. According to the Kramers-Krong relaxation between *ε*′ and *ε*″, the dielectric loss factor without DC-conductivity loss can be obtained [17]:

(3) $$\varepsilon_{\mathrm{der}}^{\prime \prime }(\omega )=-\frac{\pi }{2}[\partial \varepsilon^{\prime }(\omega )/\partial \ln(\omega )],$$

where *ε*^″^_der_ is the dielectric loss factor without DC-conductivity loss, and based on Savitzky-Golay polynomial, the differentiation of discrete dielectric spectrum data is established [18]:

(4) $$\varepsilon_{\mathrm{der}}^{\prime \prime }(\omega )=-\frac{\pi }{2}[-2\varepsilon^{\prime }(\omega q^{-2})-\varepsilon^{\prime }(\omega q^{-1})+\varepsilon^{\prime }(\omega q)+2\varepsilon^{\prime }(\omega q^{2})]\cdot \frac{1}{10\ln q},$$

where *q* is the common ratio of the frequency (*q* > 1). Figure 4b demonstrates the frequency spectrum of *ε*^″^_der_ from 20 °C to 80 °C. The covered interfacial polarization peaks are distinctly visible.

**Figure 5:**
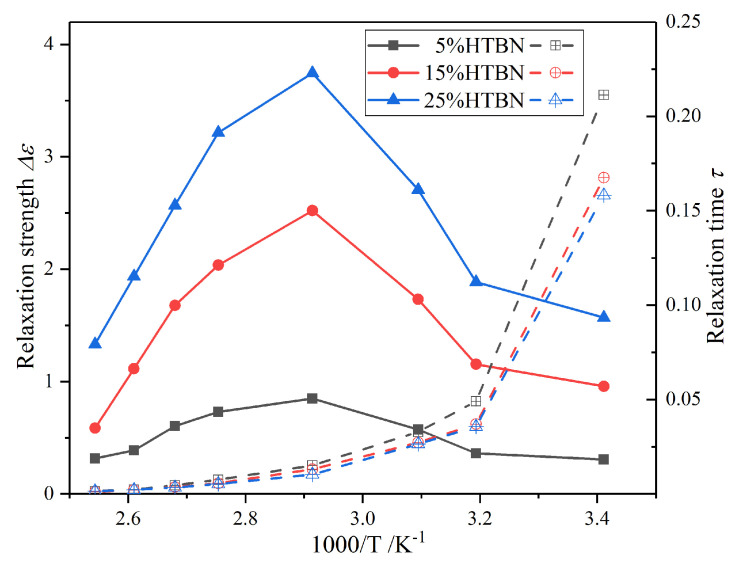
Havriliak‒Negami equation (HN-equation) fitting results of interfacial polarization of samples with different HTBN contents.

With the increase of temperature, the interfacial polarization peak moves to a higher frequency, while the relaxation strength (∆*ε*) increases firstly and then decreases. However, the polarization at 50 °C and 60 °C overlap with the *α* relaxation of epoxy resin. Thus, HN-equation fitting is carried out to quantitatively analyse the development of interfacial polarization. The temperature dependence of ∆*ε* and *τ* from 20 °C to 120 °C is manifested in Figure 5.

The ∆*ε* increases firstly and then decreases with temperature. The maximum of the relaxation strength locates at 70 °C, which is close to the glass transition temperature (*T*_g_). The influence of interfacial polarization strength is attributed to the difference of conductivity and permittivity between fillers and matrix [19]. The phase change of epoxy resin takes place near the *T*_g_, leading to the drastic increasing of conductivity and permittivity. Meanwhile, the rubber particles are in a viscous state, and the variation of parameters with temperature is smaller. Thus, the ∆*ε* increases with temperature before the *T*_g_. With the further increase of temperature, the polarization will be obstructed by the thermal motion of molecules and resulting in the reduction of ∆*ε*. In addition, the reaction range of the interfacial polarization is proportional to the HTBN contents. Therefore, the ∆*ε* in a high concentration system leads the first.

**Figure 6:**
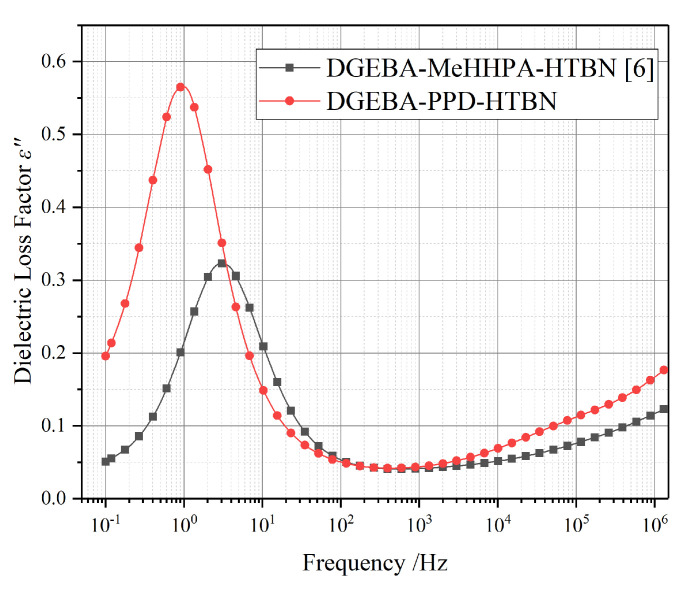
Interfacial polarization of the DGEBA-MeHHPA-HTBN and DGEBA-PPD-HTBN at 20 °C.

In Figure 6, under the same temperature and filler contents, the relaxation time of the interfacial polarization in DGEBA-MeHHPA-HTBN is slightly slower than that of DGEBA-PPD-HTBN, but the relaxation strength is 1.8 times higher. This is because it is difficult for HTBN to participate in the curing reaction of the amine formulation system and it increases the number of HTBN particles in the matrix. Moreover, due to the different glass transition temperature, the changing trend of the polarization with temperature may also be different. Therefore, the curing agent has a significant influence on the relaxation properties of epoxy/liquid rubber composites.

The relaxation time (*τ*) follows the Arrhenius laws and fitting results are shown in Table 1. Through the comparison of the samples with three different filler contents, the apparent activation energy (*E*_a_) declines with the increase of filler contents. Therefore, the interfacial polarization is easier to establish in a high filler concentration system.

Combined with Figure 1, the interfacial polarization contributes to the increase of complex permittivity. However, it is through the analysis of the temperature characteristics of the ∆*ε* and *τ* that we could control the rise of complex permittivity within the allowable range by adjusting the filler ratio. Furthermore, through the selection of liquid rubber kinds or improving the curing formulation system, the surge in ∆*ε* can be controlled away from the stable operation of the device.

### 2.5. Analysis of Dynamic Glass Transition

**Figure 7:**
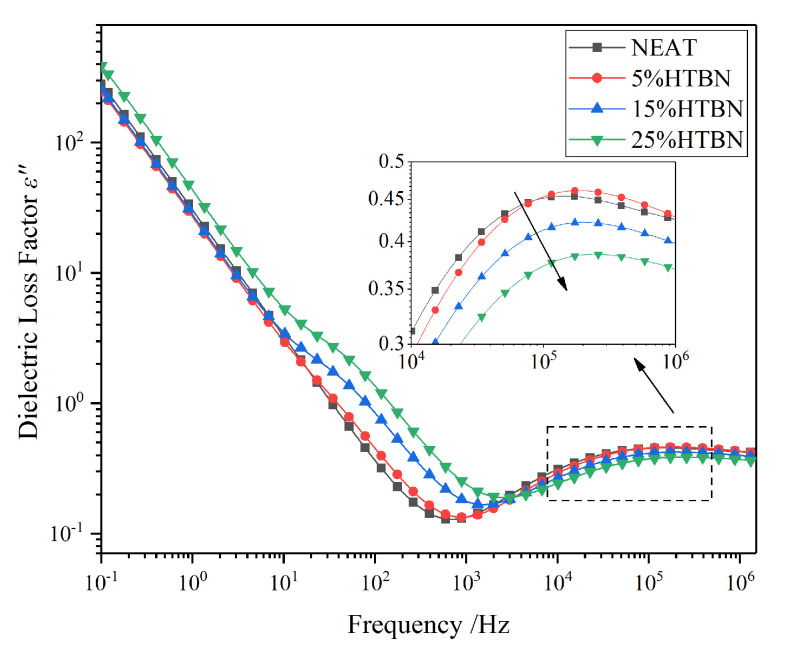
Frequency spectrum characteristics of dielectric loss factor with different HTBN contents at 90 °C.

The α relaxation of pure epoxy resin locates at the high-frequency region like a weak shoulder, as shown in Figure 7. Through the illustration, the relaxation strength of the pure epoxy resin and the sample with 5% HTBN content are much the same. However, a decline of strength with the increase of filler content is shown through the arrow. According to the scanning electron microscope (SEM) results in our previous research [20], more and larger rubber particles dispersed in high filler consternation, which causes the decrease of the number of epoxy resin molecular segments per unit volume and leads to the decline of α relaxation strength. With the increase of filler content, the free volume of the matrix enlarges, which makes the chain movement easier and the time of the relaxation shorter. Thus, the relaxation peaks move to a higher frequency.

**Figure 8:**
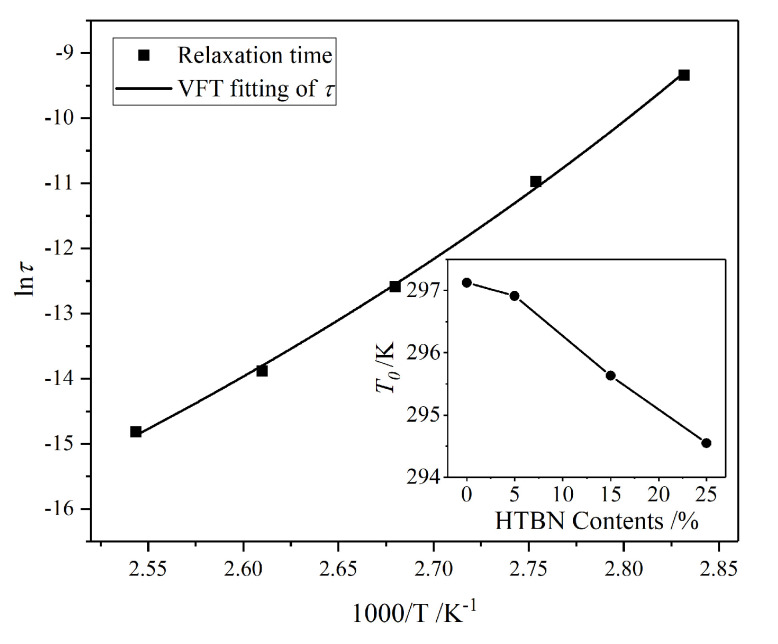
Relationship between relaxation time of epoxy resin *α* relaxation and reversed temperature. Insert figure: Vogel temperature of *α* relaxation with different HTBN contents.

The relaxation time of the *α* relaxation is extracted using HN-equation fitting, and its relationship with the reversed temperature is shown in Figure 8. The rotational orientation ability of dipolar enhances with temperature, leading to the reduction of the relaxation time.

Furthermore, there is a nonlinear relation between the natural logarithm of the relaxation time and the reversed temperature. Therefore, this relation could be described by the Vogel‒Fulcher‒Tammann equation (VFT-equation):

(5) $$\tau (T)=\tau_{\infty }\cdot \exp\left[\frac{-DT_{0}}{R(T-T_{0})}\right],$$

where *τ*(*T*) is the relaxation time when the temperature is *T*, *τ*_∞_ is the time when *T*→+∞, *D* is the constants, *R* is the universal gas constants, and *T*_0_ is the Vogel temperature, which is also called the ideal glass transition temperature.

In Table 2, the VFT-equation fitting determination coefficients indicate that the temperature dependence of the relaxation time obeys the VFT law. The *T*_g_ calculated using the Vogel temperature (*T*_0_) drops from 73.98 °C to 71.40 °C, which is consistent with the data measured using the dynamic mechanical thermal analyzer (DMA) [20]. Therefore, the two-phase structure in samples with liquid rubber speeds up the plasticizing effect of the network, leading to the slight decline of *T*_g_. In other words, the surge of complex permittivity caused by phase change will initially occur in samples with liquid rubber.

### 2.6. Analysis of Thermionic Polarization

**Figure 9:**
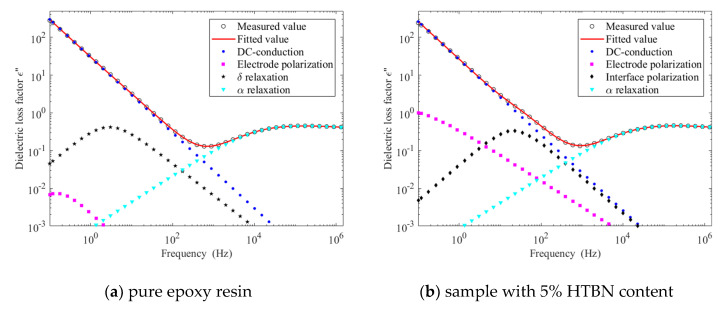
HN-equation fitting results of dielectric loss factor of pure epoxy resin and sample with 5% HTBN content at 90 °C: (**a**) pure epoxy resin; (**b**) sample with 5% HTBN content.

The HN-equation fitting results of *ε*″ at 90 °C are demonstrated in Figure 9. The relaxation peak of the pure epoxy resin appears at low frequency, which has been found in other studies [21]. This is caused by the hopping of mobile ion. On the one hand, the hopping of ion forms dipoles and establishes polarization, as in Figure 9a, which is also called thermionic polarization (δ relaxation) [22,23]. On the other hand, the directional migration of ions can form conduction current [23,24]. However, the sample with 5% HTBN content shows a distinct process in Figure 9b. Therefore, the addition of HTBN may change the hopping behaviour.

**Figure 10:**
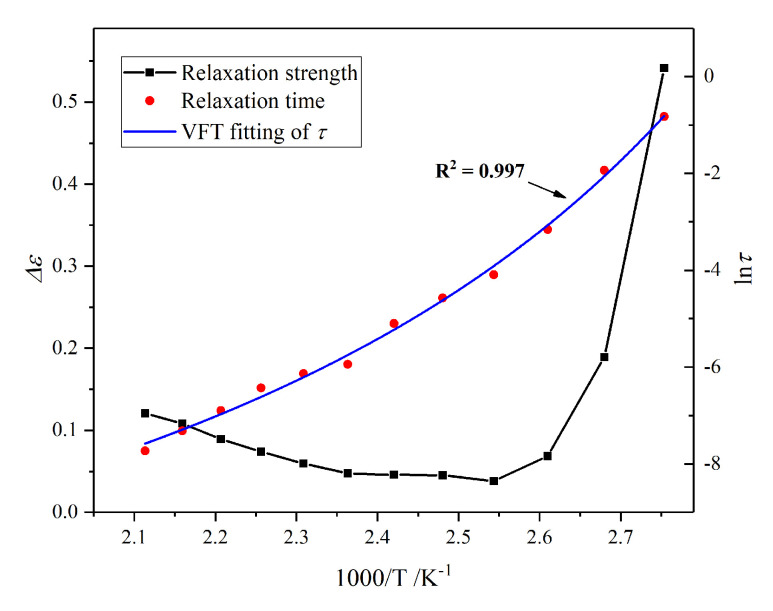
HN-equation fitting results of thermionic polarization of pure epoxy resin from 90 °C to 200 °C.

According to the HN-equation fitting, the temperature dependence of ∆*ε* and *τ* of *δ* relaxation of epoxy resin is exhibited in Figure 10. The ∆*ε* declines firstly and then magnifies with the increase of the temperature, while the *τ* keeps going down. It indicates that the thermal vibration of impurity ions is intensified and the polarization is easier to establish at a higher temperature. The *δ* process follows the VFT law, and the *T*_0_ obtained by fitting is 275.33 K.

**Figure 11:**
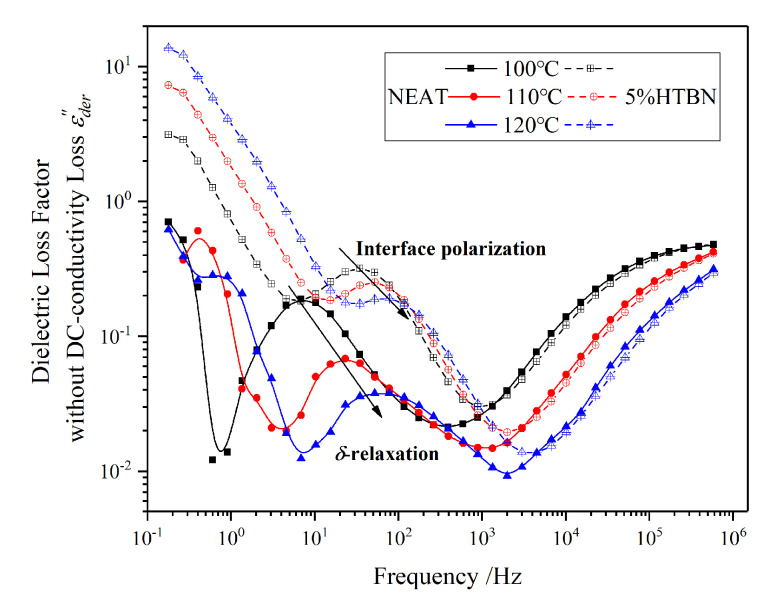
Frequency spectrum characteristics of dielectric loss factor without DC-conductivity loss from 100 °C to 120 °C.

Along with the arrows in Figure 11, the polarization of the sample with 5% HTBN content in medium frequency describes the interfacial polarization. With the increase of temperature, the peaks are close to the *δ* relaxation of pure epoxy resin. In addition, when the temperature is 120 °C, the difference between the two polarizations is almost an order of magnitude. Therefore, the motion of the impurity ions could be affected by the interfacial polarization. The bound charge accumulating on the interface between the rubber and epoxy matrix may restrain the *δ* relaxation likewise.

### 2.7. Analysis of DC-Conductivity

**Figure 12:**
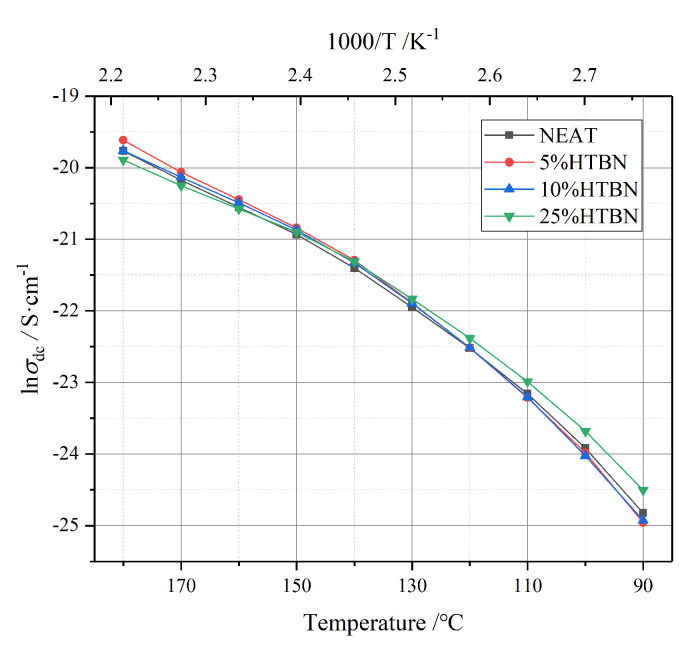
HN-equation fitting results of DC-conductivity with different HTBN contents from 90 °C to 180 °C.

Through HN-equation fitting, the temperature spectrum of DC-conductivity (*σ*_dc_) is illustrated in Figure 12. The *σ*_dc_ of all samples is directly proportional to temperature. Interestingly, the *σ*_dc_ of the different sample varies with the change rate of temperature, resulting in the intersection of four curves around 150 °C, which is consistent with the changing trend of *ε*″, and the *ε*″ above 120 °C is almost determined by the *σ*_dc_. Therefore, the *σ*_dc_ is the main factor that contributes to the variation of *ε*″ in the high-temperature region.

The temperature characteristics of the *σ*_dc_ follow the Arrhenius law. According to Figure 12, the Arrhenius equation is employed to calculate activation energy, and the fitting results are shown in Table 3. The *E*_a_ declines with increasing HTBN contents and the sample with 25% HTBN content manifests lower *E*_a_.

The factors that determine the conductivity are the concentration and the mobility of impurity ions [24,25]. The addition of HTBN introduces more impurity ions into the polymers. As previously mentioned, the interface acts as a charge collector, which could cause the distortion of the electric field and impede the migration of carriers. These two factors give rise to a change in the *σ*_dc_. Therefore, according to the intersection in Figure 12, we could infer that the former factor dominates when the temperature is below 150 °C. The density of impurity ions rises with the increase of filler content, leading to the maximum *σ*_dc_ of the sample with 25% HTBN content, and the latter dominates when the temperature is above 150 °C; the obstruction to carrier migration is intensified due to the larger interface in the sample with a high filler ratio. Thus, the samples with filler ratios of 15% and 25% present a lower DC-conductivity than the pure epoxy resin.

## 3. Materials and Methods

### 3.1. Materials

**Figure 13:**
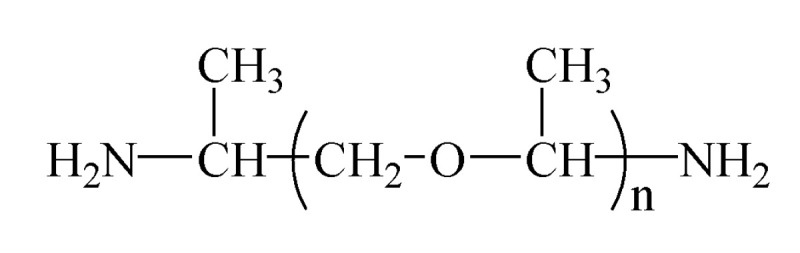
The molecular formula of polyoxide propylene diamine.

The epoxy resin used in this study is diglycidyl ether of bisphenol A (DGEBA), with an epoxy value of 3.9 mmol/g, produced by Xingchen Synthetic Material Co., Ltd., Nantong, China. The curing agent is polyoxide propylene diamine (PPD), purchased from Zhongsi Industrial Co. Ltd., Shanghai, China. The molecular formula is shown in Figure 13, and the average molecular weight (Mn) adopted in our research is 400. This kind of long-chain type amine curing agent has the effect of improving crack resistance of the samples. More importantly, this curing system initially cures at 40 °C, meeting the requirements of the coating material, and the full curing temperature is above 60 °C, which ensures the mechanical strength of the curing product at the same time. The filler for modification is HTBN, with a number average molecular weight of 2 × 10^3^, supplied from Qilong Chemical Co., Ltd., Zibo, China. The contents of the hydroxyl group is 0.6 mmol/g. All reagents are used without special treatment.

### 3.2. Preparation of Samples

The epoxy resin, curing agent and catalytic agent, with a weight ratio of 100:39:0.5 and four blends with HTBN, whose content ranges from 0% to 25%, were prepared according to the procedure below. At first, HTBN is added into the epoxy resin. The blend is mixed and pre-crosslinked at 150 °C for 1 h. Then, the blend is cooled to room temperature. After that, the curing agent is poured into the blend and mixed evenly with a low-speed agitator. The vacuum degassing treatment is carried out after mixing. The curing process is divided into pre-curing and post-curing. After the pre-curing at 40 °C, the two-phase structure of rubber particles and the epoxy matrix is initially formed. Then, to avoid uneven curing, post-curing is carried out by step heating, and the duration of 80 °C and 110 °C is extended for complete solidification. Finally, the curing process is under ambient conditions of 40 °C for 2 h, 60 °C for 2 h, 80 °C for 3 h and 110 °C for 4 h. After that, the sample is stepwise cooled before removing from the oven.

### 3.3. Properties Measure

The German Novocontrol company’s concept80 broadband dielectric spectroscopy tester was used to measure the dielectric properties. The size of the sample was 40 mm in diameter and 1 mm in thickness. Using ion sputtering, one side of the sample was coated with gold, and the other side was coated with a circle of 30 mm. The frequency range was from 10^−1^ Hz to 10^6^ Hz, and the temperature range was from −60 °C to 200 °C.

## 4. Conclusions

The dielectric relaxation behaviours of the composites are extracted and their characteristics are analysed. The existence of HTBN molecule restrains the *γ* process and introduces a new relaxation polarization and superimposes with the β relaxation of epoxy resin leading to the rise of complex permittivity in the low-temperature region. The reduction in *T*_g_ with the increase of filler content is consistent with the Vogel temperature, which is attributed to the enhancement of the plasticizing effect by the two-phase structure in the sample with liquid rubber.

Compared with the pure epoxy resin, the interfacial polarization in modified composites plays a critical role in the change of relaxation behaviours and conduction near and above *T*_g_. The variation of the interfacial polarization strength reveals the phase change process of the epoxy resin, reaching its maximum near the *T*_g_. Furthermore, the interface between the filler and matrix obstructs the motion of impurity ions, which contributes to the inhibition of thermionic polarization and the intersection of DC-conductivity in the temperature spectrum. Therefore, during the selection of liquid rubber, the temperature dependence of the strength and time of the interfacial polarization should be paid particular attention to.

## Figures and Tables

**Table 1:** Arrhenius fitting results of relaxation time of the interfacial polarization.

HTBN Contents (%)	*E*_a_ (kJ·mol^−1^)	*E*_a_ (eV)	*R* ^2^
5	44.708	0.465	0.994
15	44.292	0.461	0.993
25	43.046	0.448	0.997

***E*_a_**: apparent activation energy, ***R*^2^**: determination coefficients.

**Table 2:** Vogel‒Fulcher‒Tammann equation (VFT-equation) fitting results of relaxation time of α relaxation.

HTBN Contents (%)	ln*τ*_∞_ (s)	*T*_0_ (K)	*R* ^2^
0	−22.505	297.127	0.995
5	−22.249	296.913	0.999
15	−22.464	295.632	0.999
25	−22.505	294.547	0.999

**Table 3:** Arrhenius equation fitting results of DC-conductivity.

HTBN Contents (%)	*E*_a_ (kJ·mol^−1^)	*E*_a_ (eV)	*R* ^2^
0	75.289	0.783	0.992
5	79.444	0.826	0.990
15	77.699	0.808	0.988
25	69.056	0.718	0.989

## References

[1] Liu H., Wang G., Mai Y., Zeng Y. (2011). On fracture toughness of nano-particle modified epoxy. Compos. Part A Eng..

[2] Lowe A., Kwon O., Mai Y. (1996). Fatigue and fracture behaviour of novel rubber modified epoxy resins. Polymer.

[3] Bian X., Tuo R., Yang W., Zhang Y., Xie Q., Zha J., Lin J., He S. (2019). Mechanical, Thermal, and Electrical Properties of BN–Epoxy Composites Modified with Carboxyl-Terminated Butadiene Nitrile Liquid Rubber. Polymers.

[4] Gong Y., Zhou W., Kou Y., Xu L., Wu H., Zhao W. (2017). Heat conductive h-BN/CTPB/epoxy with enhanced dielectric properties for potential high-voltage applications. High Volt..

[5] Wang C., Li H., Zhang H., Wang H., Liu L., Xu Z., Liu P., Peng Z. (2016). Influence of addition of hydroxyl-terminated liquid nitrile rubber on dielectric properties and relaxation behavior of epoxy resin. IEEE Trans. Dielectr. Electr. Insul..

[6] Wang C., Sun Q., Lei K., Chen C., Yao L., Peng Z. (2020). Effect of Toughening with Different Liquid Rubber on Dielectric Relaxation Properties of Epoxy Resin. Polymers.

[7] Wise C.W., Cook W.D., Goodwin A.A. (2000). CTBN rubber phase precipitation in model epoxy resins. Polymer.

[8] Ramos V., Costa H., Soares V., Nascimento R. (2005). Modification of epoxy resin: A comparison of different types of elastomer. Polym. Test..

[9] Kamar N., Drzal L. (2016). Micron and nanostructured rubber toughened epoxy: A direct comparison of mechanical, thermomechanical and fracture properties. Polymer.

[10] Thomas R., Durix S., Sinturel C., Omonov T., Goossens S., Groeninckx G., Moldenaers P., Thomas S. (2007). Cure kinetics, morphology and miscibility of modified DGEBA-based epoxy resin—Effects of a liquid rubber inclusion. Polymer.

[11] Kou Y., Zhou W., Li B., Dong L., Duan Y., Hou Q., Liu X., Cai H., Chen Q., Dang Z. (2018). Enhanced mechanical and dielectric properties of an epoxy resin modified with hydroxyl-terminated polybutadiene. Compos. Part A Manuf..

[12] Huang Y., Min D., Li S., Wang X., Lin S. (2017). Dielectric relaxation and carrier transport in epoxy resin and its microcomposite. IEEE Trans. Dielectr. Electr. Insul..

[13] Kremer F., Schönhals A. (2003). Broadband Dielectric Spectroscopy.

[14] Ning X., Feng H., Zhang H., Liu P., Xiang Z., Peng Z. (2015). Dielectric properties of multi-layer epoxy resinimpregnated crepe paper composites. IEEE Trans. Dielectr. Electr. Insul..

[15] Couderc H., Frechette M., David E., Savoie S. (2013). Study of dielectric relaxation of epoxy composites containing micro and nano particles. IEEE Trans. Dielectr. Electr. Insul..

[16] Delides C.G., Vatalis A.S., Pissis P., Pethrick R. (1993). Dielectric and thermally stimulated discharge current studies of rubber-modified epoxy resins. J. Macromol. Sci. Part B Phys..

[17] Michael W., Turnhout J.V. (2012). Analysis of complex dielectric spectra. I. One-dimensional derivative techniques and three-dimensional modelling. J. Non-Cryst. Solids.

[18] Savitzky A., Golay M. (1964). Smoothing and differentiation of data by simplified least squares procedures. Anal. Chem..

[19] Suo C., Li Z., Zheng H., Sun Y. (2017). Dynamic characteristics analysis on interface polarization and depolarization of nonlinear double-layered dielectrics. IEEE Trans. Dielectr. Electr. Insul..

[20] Wang C., Jia J., Sun Q., Zhao L., Jia R., Peng Z. Thermal and Electrical Properties of Room Temperature Curing Epoxy Resin Modified with Hydroxyl-terminated Nitrile Rubber. Proceedings of the 2019 2nd International Conference on Electrical Materials and Power Equipment (ICEMPE).

[21] Min D., Li S., Hirai N., Ohki Y. (2016). Dielectric spectroscopic analysis of degradation in ethylene-propylene-diene copolymer. IEEE Trans. Dielectr. Electr. Insul..

[22] Kao K.C. (2004). Dielectric Phenomena in Solids.

[23] Sidebottom D.L. (2009). Colloquium: Understanding ion motion in disordered solids from impedance spectroscopy scaling. Rev. Mod. Phys..

[24] Dyre J.C., Thomas B.S. (2000). Universality of AC Conduction in Disordered Solids. Rev. Mod. Phys..

[25] Sha Y., Zhou Y., Nie D., Wu Z., Deng J. (2014). A study on electric conduction of transformer oil. IEEE Trans. Dielectr. Electr. Insul..

